# Evidence of a novel α-synuclein strain isolated from a Parkinson’s disease with dementia patient sample

**DOI:** 10.1186/s40478-025-02093-x

**Published:** 2025-08-18

**Authors:** Sara A. M. Holec, Jisoo Lee, Chase R. Khedmatgozar, Marcelina J. Wojewska, Abby Oehler, Glenda M. Halliday, Steve M. Gentleman, Amanda L. Woerman

**Affiliations:** 1 Department of Microbiology, Immunology, and Pathology, Prion Research Center, Colorado State University, 300 West Lake Street, Fort Collins, CO 80521 USA; 2 Institute for Neurodegenerative Diseases, Weill Institute for Neurosciences, University of California, San Francisco, CA USA; 3 Department of Brain Sciences, Imperial College London, London, UK; 4 Brain and Mind Centre & Faculty of Medicine and Health School of Medical Sciences, University of Sydney, Sydney, Australia; 5 Faculty of Medicine, School of Medical Science, University of New South Wales, Kensington, Australia; 6 Neuroscience Research Australia, Randwick, Australia; 7 Present Address: Sangamo Therapeutics, Brisbane, CA USA

**Keywords:** Lewy body diseases, Neurodegenerative disease, Strain interactions, Synucleinopathies

## Abstract

Multiple system atrophy (MSA) and the Lewy body diseases (LBDs) are caused by α-synuclein misfolding into distinct conformations, or strains, with unique biological properties. MSA patient samples readily transmit disease following intracranial (i.c.) inoculation into humanized mice, whereas LBD samples typically do not. Unexpectedly, we identified one LBD patient sample that, following i.c. inoculation, transmitted neurological disease to four out of six mice over an extended incubation period. In light of these unexpected results, we sought to identify the α-synuclein strain responsible for disease onset. Using immunohistochemistry, we identified both Lewy bodies and oligodendroglial inclusions with a glial cytoplasmic inclusion-like appearance in the substantia nigra of the patient sample. To determine if these glial inclusions were due to the presence of low titer MSA α-synuclein in the sample, we performed a secondary passage of two terminal mouse brains from the primary passage and found that the humanized mice developed disease with a shortened incubation period. Unexpectedly, using our panel of mutant α-syn140–YFP cells to analyze the primary and secondary passage samples showed that the strain isolated in the in vivo studies has unique biological properties compared to the MSA and LBD strains. These data suggest that the oligodendroglial pathology in the LBD patient sample was not caused by MSA co-pathology, and provide evidence for the isolation of a novel distinct α-synuclein strain.

## Introduction

The misfolding and accumulation of aggregated α-synuclein within the central nervous system is the underlying cause of a group of neurodegenerative diseases known as synucleinopathies. Notably, while α-synuclein inclusions feature in each of these distinct clinical disorders, the hallmark neuropathology present at the time of autopsy is used to subcategorize the underlying disease. For example, multiple system atrophy (MSA) is characterized by the presence of α-synuclein accumulating into neuronal and glial cytoplasmic inclusions (GCIs) [1–3]. By comparison, the formation of Lewy bodies (LBs) and Lewy neurites characterizes the Lewy body diseases (LBDs), which include Parkinson’s disease (PD), dementia with Lewy bodies (DLB), and Parkinson’s disease with dementia (PDD) [4, 5].

The distinct neuropathological lesions seen in patients with MSA and LBDs are hypothesized to be caused by unique misfolded α-synuclein fibril conformations, or strains. The strain hypothesis was first proposed to explain how the cellular prion protein (PrP^C^) can cause an array of diseases across multiple species when the protein misfolds into the pathogenic PrP scrapie (PrP^Sc^) conformation [6–9]. In addition to the biochemical and biological data supporting the strain hypothesis, structural studies now confirm that each prion disease is caused by PrP^Sc^ misfolding into a distinct fibril structure [10–16]. Similar findings for other misfolding proteins, including α-synuclein, have expanded the application of the strain hypothesis beyond PrP^Sc^ to explain the clinical heterogeneity seen across many neurodegenerative diseases.

Research investigating the role of α-synuclein strain biology in human disease has largely relied on the TgM83 mouse model, which was originally reported in 2002 by Giasson et al. [17]. Homozygous TgM83^+/+^ mice express human α-synuclein with the PD-associated A53T mutation and develop a spontaneous synucleinopathy at ~1 year of age. In 2012, Thierry Baron’s laboratory showed that intracranial (i.c.) inoculation of brain homogenates from symptomatic TgM83^+/+^ mice into young, unaffected TgM83^+/+^ animals induces an earlier onset of disease [18]. Following these studies, Joel Watts and colleagues demonstrated that symptomatic TgM83^+/+^ brain homogenates, as well as MSA patient samples, can also transmit neurological disease to hemizygous TgM83^+/-^ mice, which typically remain asymptomatic through ~2 years of age [19]. By comparison, TgM83^+/-^ mice inoculated with 6 PD patient samples remained asymptomatic for > 1 year [20]. This finding was bolstered by our studies using HEK293T α-synuclein biosensor cell lines, which readily propagate MSA patient samples but are unable to support replication of LBD α-synuclein [21]. Notably, the only evidence of partial transmission following i.c. inoculation with PD patient samples occurred using samples that were sonicated prior to injection, likely fragmenting fibrils to enable the altered transmissibility observed [22]. Altogether, these data provide overwhelming biological evidence for the presence of distinct α-synuclein strains in MSA and LBD patients. Indeed, stemming from advances made in cryo–electron microscopy, it is now known that α-synuclein misfolds into distinct fibril conformations in MSA and LBD patients [23, 24].

As part of our ongoing efforts to investigate the biological consequences of these distinct α-synuclein fibril structures, we unexpectedly found one PDD human patient sample that transmitted clinical disease to four out of six TgM83^+/-^ mice over 533 dpi. Secondary passage of the PDD patient sample resulted in transmission with a reduced incubation period, though the total incubation time was still slightly longer than is typically observed following MSA i.c. inoculation. Comparing the α-synuclein strain biology present in primary and secondary passage samples using our robust panel of mutant α-syn140–YFP biosensor cell lines [25], we found that the isolated strain is distinct from both the MSA [25] and PDD strain profiles [21, 26]. Moreover, this novel strain is highly promiscuous, with an increasing capacity to replicate using α-synuclein monomer containing multiple mutations as substrate after each passage. These findings raise important implications for potential interactions between multiple strains of a single protein in neurodegenerative diseases, including the potential for strain interference.

## Materials and methods

### Human tissue samples

Control patient (C2) tissue was provided by Martin Ingelsson (Uppsala University). Frozen brain tissue samples from two MSA (MSA2 and MSA16) patient sample were provided by the Massachusetts Alzheimer’s Disease Research Center. Fresh tissue was bisected longitudinally with one half coronally sectioned prior to rapid freezing and the other half fixed in 10% neutral buffered formalin prior to sectioning. Histological evaluation was performed using Luxol fast blue and hematoxylin and eosin (H & E) staining on a set of blocked regions representative for a variety of neurodegenerative diseases. Tissue from three MSA (MSA3, MSA4, and MSA5) patient samples were provided by the Sydney Brain Bank. For these brains, one hemisphere was randomly designated for fresh dissection and the other was fixed for at least two weeks in 15% buffered formalin prior to sectioning. Standard neuropathological assessment was performed on H & E-stained sections. Tissue from one PDD (PDD1) patient sample was provided by the Parkinson’s UK (UKPD) Brain Bank. Patient brain samples (7-µm sections) were deparaffinized and rehydrated before treatment with 0.3% hydrogen peroxide for 30 min to block endogenous peroxide activity. Epitope retrieval was performed by immersing the sections in 100% formic acid for 10 min. Sections were blocked with 10% normal horse serum prior to overnight incubation at 4 °C with the anti-α-synuclein antibody clone 42 (1:1,000; BD Biosciences). Staining was visualized using the ImmPRESS horseradish peroxidase (HRP) horse anti-mouse IgG polymer detection kit (Vector Laboratories) followed by the ImmPACT DAB substrate HRP kit (Vector Laboratories). Sections were then counterstained with Mayer’s hematoxylin, dehydrated, cleared, and mounted with DPX mounting medium. Brightfield images were acquired with a DS-Fi2 Digital Camera attached to a Nikon Eclipse 50i microscope using a DS-U3 Digital Camera Controller and NIS-Elements image acquisition software. All samples were anonymized. Demographic information about the patient samples used is included in Table 1.

**Table 1:** Patient sample information

Sample	Brain Region	Diagnosis	Age at Death	Sex	Brain Bank
C2	Frontal cortex	None	88	F	Uppsala University
MSA2	Midbrain	MSA	68	M	MADRC^a^
MSA3	Pons	MSA	61	M	Sydney Brain Bank
MSA4	Pons	MSA	82	M	Sydney Brain Bank
MSA5	Pons	MSA	61	M	Sydney Brain Bank
MSA16	Midbrain	MSA	66	F	MADRC^a^
PDD1	Substantia nigra & basal ganglia	PDD	82	M	UKPD^b^

^a^MADRC, Massachusetts Alzheimer’s Disease Research Center

^b^UKPD, Parkinson’s UK Brain Bank

### Mouse inoculations

Animals were housed in AAALAC-accredited ABSL-2 conditions with a standard 12-h light/dark cycle. TgM83^+/+^ male mice [17] were bred with B6C3F1 female mice, both purchased from Jackson Laboratory, to generate the TgM83^+/-^ mice used in this study. All mice were group housed unless separation was necessary for health reasons.

For inoculation studies, fresh-frozen human or mouse-passaged tissue was homogenized in calcium- and magnesium-free 1X Dulbecco’s phosphate buffered saline (DPBS) using the Omni Tissue Homogenizer (Omni International) to create a 10% (wt/vol) homogenate. Human patient samples C2, PDD1, and MSA16 were diluted in 1X DPBS to create a 1% (wt/vol) homogenate in 5% (wt/vol) bovine serum albumin in 1X DPBS. Mouse-passaged homogenates and the additional MSA samples were diluted to 1 mg/mL in 1X DPBS. Six-to-eight-week-old TgM83^+/-^ mice were anesthetized with isoflurane prior to i.c. injections using 30 µL of 1 mg/mL brain homogenate into the right parietal lobe. Mice were assessed three times each week for the onset of neurological signs based on standard prion disease criteria [27] and were euthanized either following the onset of progressive neurological signs or at experimental endpoint (365 days postinoculation [dpi] for C2 inoculation and 380 dpi for mouse-passaged control inoculation). More specifically, mice were evaluated on a 0–3 scale for hindlimb clasping and forepaw strength. The humane endpoint was defined by a consistent score of 3 on both assessments along with the onset of at least one additional clinical sign (e.g., ataxia, kyphosis, difficulty righting, tail rigidity, loss of bladder control as exhibited by urine staining, hindlimb paralysis) to confirm onset of progressive disease. Following euthanasia, the brain was removed and bisected down the midline. One half was fixed in formalin to visualize α-synuclein neuropathology, and the other half was frozen for biochemical and biological analyses.

### α-synuclein prion quantification assay

Pathogenic α-synuclein aggregates were isolated from human and mouse brain homogenates using phosphotungstic acid (PTA; Sigma), as reported previously [21]. To quantify pathogenicity of the protein aggregates isolated from C2 and PDD1 substantia nigra (SN) human patient samples, as well as primary passage or C2, PDD1, and MSA16, protein pellets were resuspended and diluted in 1X DPBS (1:10) before incubating with Lipofectamine 2000 (Thermo Fisher Scientific) for 1.5 h. Using HEK293T cell lines expressing mutant human α-synuclein fused to yellow fluorescent protein (YFP) and assay conditions that have been previously reported [28], the C2 and PDD1 human patient samples were tested for their ability to replicate in cells expressing the E46K and A53T single mutations as well as the double A30P, A53T mutations. Primary passage samples were initially tested in cells expressing the A53T mutation only. Samples were incubated with the cells for 4 d prior to imaging using the IN Cell Analyzer 6000 (GE Healthcare). Fluorescein isothiocyanate (FITC) and 4’,6-diamidino-2-phenylindole (DAPI) channels were used to collect images from five different regions in each of six technical replicate wells. Images were analyzed via the IN Cell Developer software (GE Healthcare) with a custom-built algorithm using size and fluorescence thresholds to quantify the intracellular aggregates in living cells, represented as total fluorescence per cell (× 10^3^ arbitrary units [A.U.]). Further analysis of samples from symptomatic mice following the PDD1 primary passage, all secondary passage samples, MSA patient samples, and primary passage of MSA patient samples MSA2, MSA3, MSA4, and MSA5, was performed using a larger panel of α-synuclein–YFP cell lines as previously reported [25, 29]. The isolated protein aggregates were resuspended and diluted in 1X DPBS using conditions optimized for each cell line (A30G, 1:5; E46K, 1:10; G51D, 1:6; A53E, 1:10; A53T, 1:28; V55Y, 1:20; V66F, 1:10; V74P, 1:10; and K80E, 1:20) before the addition of Lipofectamine 2000. After incubating samples with each cell line for 4 d, cells were imaged using the Lionheart FX automated microscope (Agilent BioTek) to capture both DAPI and YFP images from four regions of interest for each of six technical replicate wells. Custom algorithms based on aggregate size and fluorescence intensity optimized to each cell line were built using BioTek Gen5 software (Agilent BioTek). These algorithms were used to analyze each image to quantify intracellular aggregates in living cells, reported as total fluorescence per cell (× 10^5^ A.U.). For all samples, regardless of imaging system, regions of interest from each well were combined to calculate one value per well. The values of the six technical replicates were then used to determine the average and standard deviation for each sample. For statistical comparisons, data were only directly compared if collected on the same imaging system.

### Mouse immunohistochemistry and neuropathology

Formalin-fixed mouse half-brains were coronally sectioned, processed through graded alcohols, xylene, and paraffin, and embedded, as described previously [30]. Tissue was cut into 8-µm sections, mounted, and deparaffinized prior to heat-mediated antigen retrieval in citrate buffer (0.1 *M*, pH 6) for 20 min. Slides were blocked in 10% (vol/vol) normal goat serum before overnight incubation at room temperature with primary antibodies EP1536Y (1:1,000; Abcam) and glial fibrillary acidic protein (GFAP; 1:500; Abcam). Primary antibody staining was detected using secondary antibodies conjugated to Alexa Fluor 488 and 594, respectively (1:500; Thermo Fisher Scientific). Nuclei were labeled with Hoechst 33,342 (1:5,000; Thermo Fisher Scientific). Slides from primary passage experiments were imaged using the Zeiss Axio Scan.Z1 and were analyzed using the ZEN Software (Zeiss). Slides from secondary passage experiments were imaged using the Lionheart FX with the 10× objective and were analyzed using the BioTek Gen5 software package. α-Synuclein pathology was quantified by drawing regions of interest around the caudoputamen (Cd), hippocampus (HC), piriform cortex & amygdala (Pir), thalamus (Thal), hypothalamus (HTH), midbrain (Mid), and pons after setting a pixel intensity threshold using positive and negative control slides. The percentage of positive pixels in each measured region was averaged across all mice in an experimental group. The Leica DMi8 inverted fluorescent microscope (Leica Microsystems) was used to capture representative images of phosphorylated α-synuclein pathology from secondary passage of PDD1, which were then processed using LAS X software (Leica Microsystems). Neuropathology data were not directly compared unless imaged using the same microscope.

### Statistical analysis

Data were analyzed using GraphPad Prism software (version 10) and are presented as mean ± standard deviation. Cell assay data comparing C2 and PDD1 infection were analyzed using an unpaired *t* test. Kaplan–Meier curves were analyzed using a log-rank Mantel–Cox test. Cell assay data comparing the primary passage experiments were analyzed using a one-way analysis of variance (ANOVA) followed by a Tukey’s multiple comparisons post hoc test. Neuropathology data from primary and secondary passage experiments were analyzed using a two-way ANOVA followed by a Dunnett’s multiple comparisons post hoc test. Cell assay comparisons between primary and secondary passage of C2 and PDD1 were analyzed using an unpaired *t* test. Significance was determined with a *P* value < 0.05.

## Results

### Human patient sample PDD1 unexpectedly induces clinical disease in TgM83^+/-^ mice

**Fig. 1:**
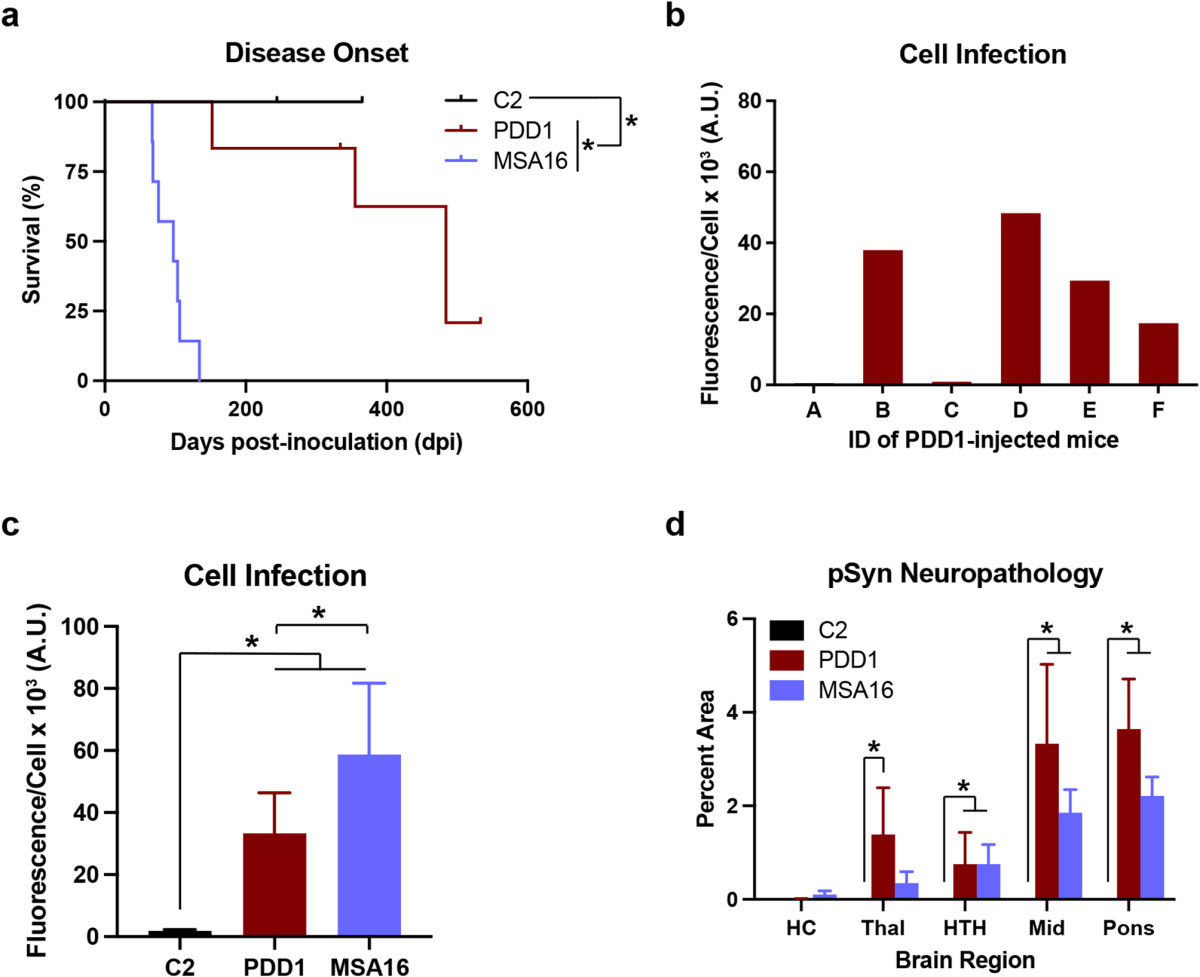
Human patient sample PDD1 induces neurological disease in TgM83^+/-^ mice following intracranial inoculation. (**a**) Kaplan–Meier plot showing disease onset in TgM83^+/-^ mice intracranially (i.c.) inoculated with 30 µL of 1% (wt/vol) brain homogenate of human patient samples C2 (*n* = 9), PDD1 (*n* = 6), and MSA16 (*n* = 7). Data for C2- [28] and MSA16-inoculated mice [33] were previously reported. C2-inoculated mice were asymptomatic > 365 days postinoculation (dpi). Mice injected with PDD1 developed neurological signs 390 ± 141 dpi with four of six mice developing clinical signs, and mice inoculated with MSA16 developed clinical disease 93 ± 24 dpi. (**b**) Quantification of α-synuclein prion infectivity in α-syn140*A53T-YFP cells using protein aggregates isolated from individual TgM83^+/-^ mouse brains (Mice A-F) following primary passage of PDD1. Each sample was tested in six replicate wells. (**c**) Quantification of α-synuclein prion infectivity in α-syn140*A53T-YFP cells using protein aggregates isolated from TgM83^+/-^ mouse brains following primary passage of C2 (*n* = 10), PDD1 (*n* = 4), and MSA16 (*n* = 7) patient samples (× 10^3^ A.U.). Data from C2- [28] and MSA16-inoculated mice [33] were previously reported. (**d**) Quantification of phosphorylated α-synuclein neuropathology (EP1536Y primary antibody; 1:1,000; Abcam) in the hippocampus (HC), thalamus (Thal), hypothalamus (HTH), midbrain (Mid), and pons of TgM83^+/-^ mice following i.c. injection with C2 (*n* = 8), PDD1 (*n* = 4), or MSA16 (*n* = 7) patient samples. Neuropathology from C2- [28] and MSA16-inoculated mice [33] were previously reported. Data shown as mean ± standard deviation. *P < 0.05

Although two groups have shown that LBD inoculations in TgM83^+/-^ mice induce α-synuclein neuropathology with subclinical disease [31, 32] and one report has shown partial transmission with sonicated PD patient samples [22], MSA is the only human synucleinopathy shown to transmit clinical disease to mouse models of synucleinopathy [19, 20]. Similarly, using our cell-based assay, we previously reported that PD, PDD, and DLB patient samples, including sample PDD1, were unable to infect cells expressing the A53T mutation, whereas MSA patient samples could readily replicate in the same cell line [21]. Consistent with these previous findings, we were unable to propagate α-synuclein prions isolated from patient sample PDD1 using cell lines expressing additional familial α-synuclein LBD mutations (E46K and the combination of A30P, A53T; Table 2). Based on these data and our previous efforts to transmit LBD patient samples to TgM83^+/-^ mice [20], we were surprised to find that i.c. inoculation of patient sample PDD1 into TgM83^+/-^ animals induced neurological signs in 390 ± 141 dpi with an incomplete attack rate (Fig. 1a; Table 3). This incubation period was substantially longer and significantly different (*P* < 0.05) than previously published incubation periods for MSA, including transmission of MSA16 to TgM83^+/-^ mice (93 ± 24 dpi) [33]. Importantly, as previously reported [28], mice inoculated with control patient sample C2 remained asymptomatic through 365 dpi (Fig. 1a). Notably, control i.c. inoculations are typically euthanized ~1 year post-injection as longer incubation periods were not previously detected, as was seen here.

**Table 2:** Biosensor cell infection data testing patient sample PDD1

Mutation	C2^a^ (Ave ± SD × 10^3^ A.U.)	PDD1 (Ave ± SD × 10^3^ A.U.)	*P* value
E46K	0.6 ± 0.2	0.5 ± 0.1	0.19
A53T	1.4 ± 0.2	3.3 ± 0.6	0.0004
A30P, A53T	5.1 ± 1.8	1.7 ± 1.1	0.004

^a^Data published previously in [28]

**Table 3:** Incubation periods for primary and secondary passage of patient sample PDD1

	Primary Transmission				Secondary Transmission	
Sample	Incubation Period^a^	n/n_0_^b^	Sample	Incubation period^c^	Incubation Period^a^	n/n_0_^b^
PDD1	390 ± 141	4/6	Mouse B	152	124 ± 32	8/8
			Mouse E	484	157 ± 35	7/8

^a^Average ± standard deviation in days postinoculation (dpi)

^b^Number of symptomatic animals (n)/number of mice inoculated (n_0_)

^c^Incubation period of the mouse brain used as inoculum for secondary transmission

Following the onset of clinical signs or terminal endpoints of the study, mouse brains were bisected down the midline with one half frozen for biochemistry and one half fixed in formalin for histology. We first evaluated the infectivity of α-synuclein prions isolated from the brains of symptomatic primary passage TgM83^+/-^ mice in the α-syn140*A53T–YFP reporter cell assay (Fig. 1b). Having previously shown that the α-syn140*A53T–YFP cell assay can determine relative α-synuclein prion quantities, or titers, in individual samples [20, 33], we first evaluated the relative titer of pathogenic α-synuclein present in all mice inoculated with PDD1. While brains collected from the two asymptomatic animals contained no detectable α-synuclein prions (Mouse A, 0.5 ± 0.3 × 10^3^ A.U.; Mouse C, 0.9 ± 0.4 × 10^3^ A.U.), the brains from the four symptomatic animals contained varying levels of pathogenic α-synuclein (Mouse B, 38 ± 11 × 10^3^ A.U.; Mouse D, 48 ± 11 × 10^3^ A.U.; Mouse E, 29 ± 9.9 × 10^3^ A.U.; Mouse F, 17 ± 4.3 × 10^3^ A.U.; Fig. 1d). We then compared inoculation groups from the primary passage using the A53T cell line (Fig. 1c). Brains from terminal C2-injected mice contained no detectable pathogenic α-synuclein (1.8 ± 0.5 × 10^3^ A.U.), whereas brains from MSA16-injected animals (59 ± 23 × 10^3^ A.U.) showed significant infection compared to control (*P* < 0.0001; Fig. 1c). The MSA16 [33] and C2 data [28] were previously published elsewhere. In comparison with the MSA16 samples, PDD1-injected mice showed lower infection (33 ± 13 × 10^3^ A.U.), however, these levels were still increased compared to C2-injected animals (*P* = 0.0043; Fig. 1c).

Finally, we evaluated the neuropathology in the mice by quantifying the deposition of α-synuclein phosphorylated at Ser129. When compared to previously reported C2-inoculated animals [28], both PDD1- and MSA16-injected symptomatic mice developed pathology throughout the hindbrain, with significant neuropathology in the hypothalamus (HTH; *P* < 0.05), as well as midbrain (Mid) and pons (*P* < 0.0001; Fig. 1d). The PDD1 patient sample also induced significant inclusions in the thalamus (Thal; *P* < 0.0001). MSA16-induced neuropathology was also reported previously [33]. Despite the initial lack of transmissibility to α-syn140*A53T–YFP cells, these data clearly demonstrate transmission of pathogenic α-synuclein to TgM83^+/-^ mice following i.c. inoculation of patient sample PDD1.

### Patient sample PDD1 contains Lewy bodies with oligodendroglial pathology

To understand why patient sample PDD1 unexpectedly induced clinical disease in four of the six inoculated TgM83^+/-^ mice, we revisited the patient’s autopsy report. This patient, who developed neurological signs at 65 years old, exhibited a typical progressive course of disease over 18 years with a prominent gait disorder and rigidity. No atypical features were noted; however, several autonomic signs were reported including orthostatic hypotension, syncope, dysphagia, and dysarthria. Consistent with the PDD diagnosis, the individual suffered from confusion, memory loss, hallucinations, and paranoid delusions. Throughout the course of disease, the patient was treated with Sinemet preparations, cabergoline, bromocriptine, pergolide, and selegiline. While no medical records remain available to confirm that the patient was responsive to DOPA treatment, the use of Sinemet preparations over the 18 year course of disease is consistent with the clinical presentation of a LBD.

**Fig. 2:**
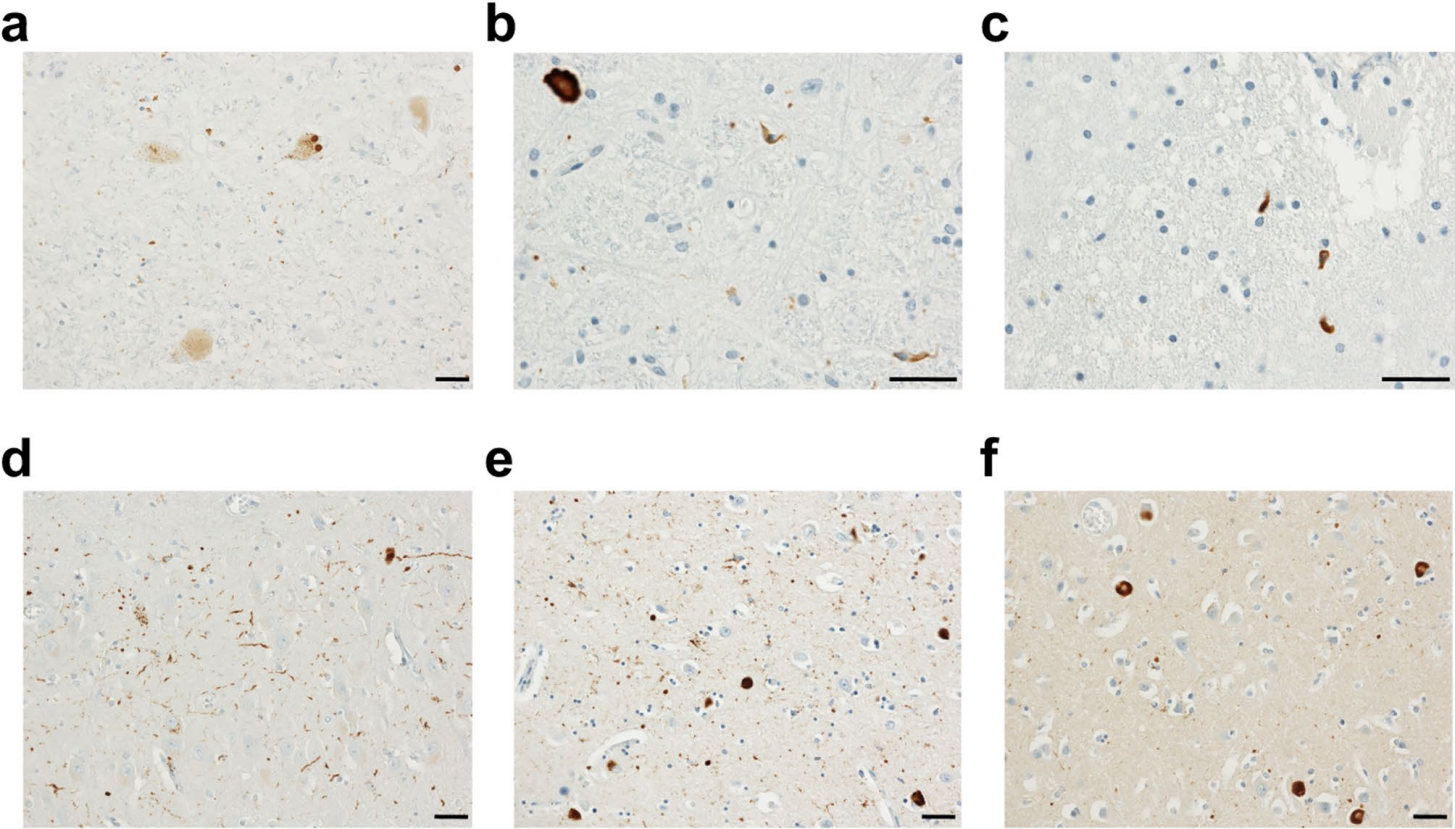
α-Synuclein immunostaining in case PDD1. (**a**) Lewy bodies in the few remaining pigmented substantia nigra neurons, together with glial staining. (**b**) Inclusions resembling glial cytoplasmic inclusions in the medulla and (**c**) the white matter of the cerebellum. (**d**) Lewy neurites in the CA2 sector of the hippocampus. (**e**) Lewy bodies, Lewy neurites, and glial staining in the amygdala. (**f**) Numerous Lewy bodies in the cingulate cortex. Images taken at 20X and 40X magnification. Scale bar, 50 μm

Re-examination of the diagnostics slides from this case revealed LBs in the few remaining pigmented substantia nigra neurons together with some oligodendroglial staining (Fig. 2a). Similar oligodendroglial staining was seen in the pontine and medullary sections (Fig. 2b) and in the white matter of the cerebellum (Fig. 2c). The hippocampus has scattered LBs and characteristic Lewy neurites in the CA2 sector (Fig. 2d). The amygdala had numerous LBs, Lewy neurites, and glial staining (Fig. 2e). Sparse oligodendroglial staining was seen in the striatal section. Scattered LBs were present in the superior frontal gyrus but they were far more numerous in the cingulate cortex (Fig. 2f). The assessment of all the α-synuclein staining was consistent with Neurocortical Lewy pathology according to the international Lewy Pathology Consensus Criteria [34], Braak stage 6. The NIA-AA: Alzheimer’s Disease Neuropathologic Change: A1, B1, C1: showed low AD neuropathologic change. This pathology is consistent with the clinical diagnosis of PDD, but the presence of oligodendroglial inclusions and the autonomic clinical signs led us to posit that the initial disease transmission seen in TgM83^+/-^ mice following i.c. injection with patient sample PDD1 was due to the presence of a low-titer MSA strain, rather than transmission of PDD to the animals. If this were the case, we would expect to find a shortened incubation period following secondary transmission to TgM83^+/-^ mice, consistent with the emergence of a low-titer strain upon primary passage.

### Secondary passage of patient sample PDD1 results in a shortened incubation period with increased neuropathology

**Fig. 3:**
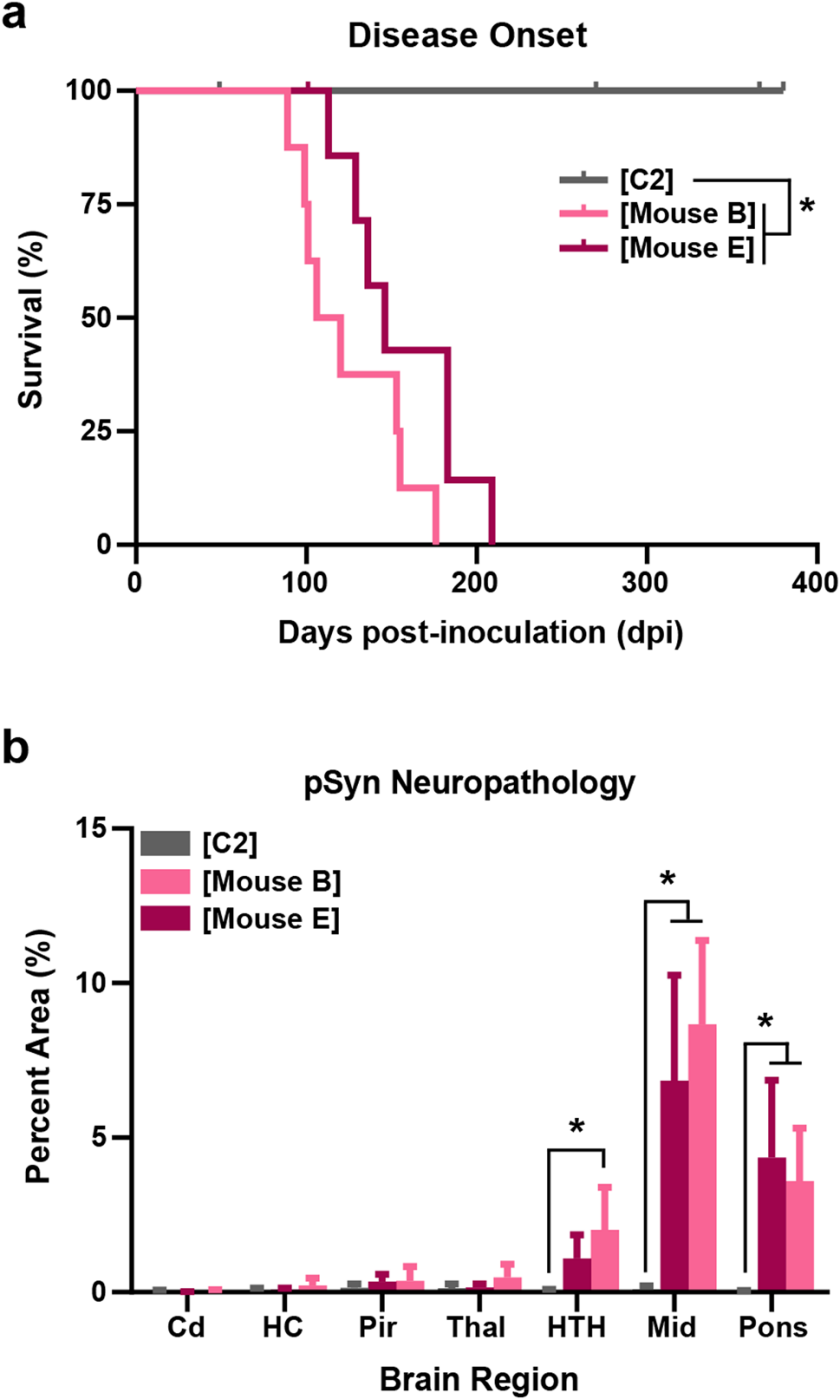
Serial passage of patient sample PDD1 results in a shortened incubation period. TgM83^+/-^ mice were intracranially (i.c.) inoculated with 30 µL of brain homogenate (standardized to 1 mg/mL total protein) of mouse-passaged C2 ([C2], gray) or mouse-passaged PDD1 from two individual mice ([Mouse B], light pink; [Mouse E], dark pink). (**a**) Kaplan–Meier plot showing mice inoculated with [C2] sample (*n* = 10) were asymptomatic through 380 days postinoculation (dpi). Data previously reported [29]. By comparison, mice injected with [Mouse B] (*n* = 8) and [Mouse E] (*n* = 8) developed neurological signs in 125 ± 32 dpi and 157 ± 35 dpi, respectively. (**b**) Quantification of phosphorylated α-synuclein pathology (EP1536Y primary antibody; 1:1,000) in the caudate (Cd), hippocampus (HC), piriform cortex and amygdala (Pir), thalamus (Thal), hypothalamus (HTH), midbrain (Mid), and pons following i.c. inoculation with [C2] (*n* = 9), [Mouse B] (*n* = 8), or [Mouse E] (*n* = 8) samples. Data shown as mean ± standard deviation. *P < 0.05

To test the hypothesis that low-titer MSA was present in the substantia nigra of patient PDD1, we performed secondary transmission studies using brain homogenate (30 µL standardized to 1 mg/mL total protein) from two different symptomatic mice selected from the PDD1 primary transmission study (Mice B and E in Fig. 1b) along with mouse-passaged C2. Control-injected mice were asymptomatic through 380 dpi, as reported previously [29], whereas the secondary passage of Mouse B (denoted as [Mouse B]) induced disease in 124 ± 32 dpi and Mouse E (denoted as [Mouse E]) transmitted clinical signs in 157 ± 35 dpi (*P* < 0.0001; Fig. 3a; Table 3). Notably, while these incubation periods are closer to the MSA16 incubation period (93 ± 24 dpi), they were still longer than is typically seen for MSA. Moreover, while the secondary passage of PDD1 induced neurological signs consistent with MSA inoculation, including loss of forepaw strength, hindlimb clasping, and paralysis, we consistently observed balance deficits and an abnormal gait in mice inoculated with homogenate from both Mouse B and Mouse E. These unusual signs were not observed in the mice inoculated with mouse-passaged MSA.

**Fig. 4:**
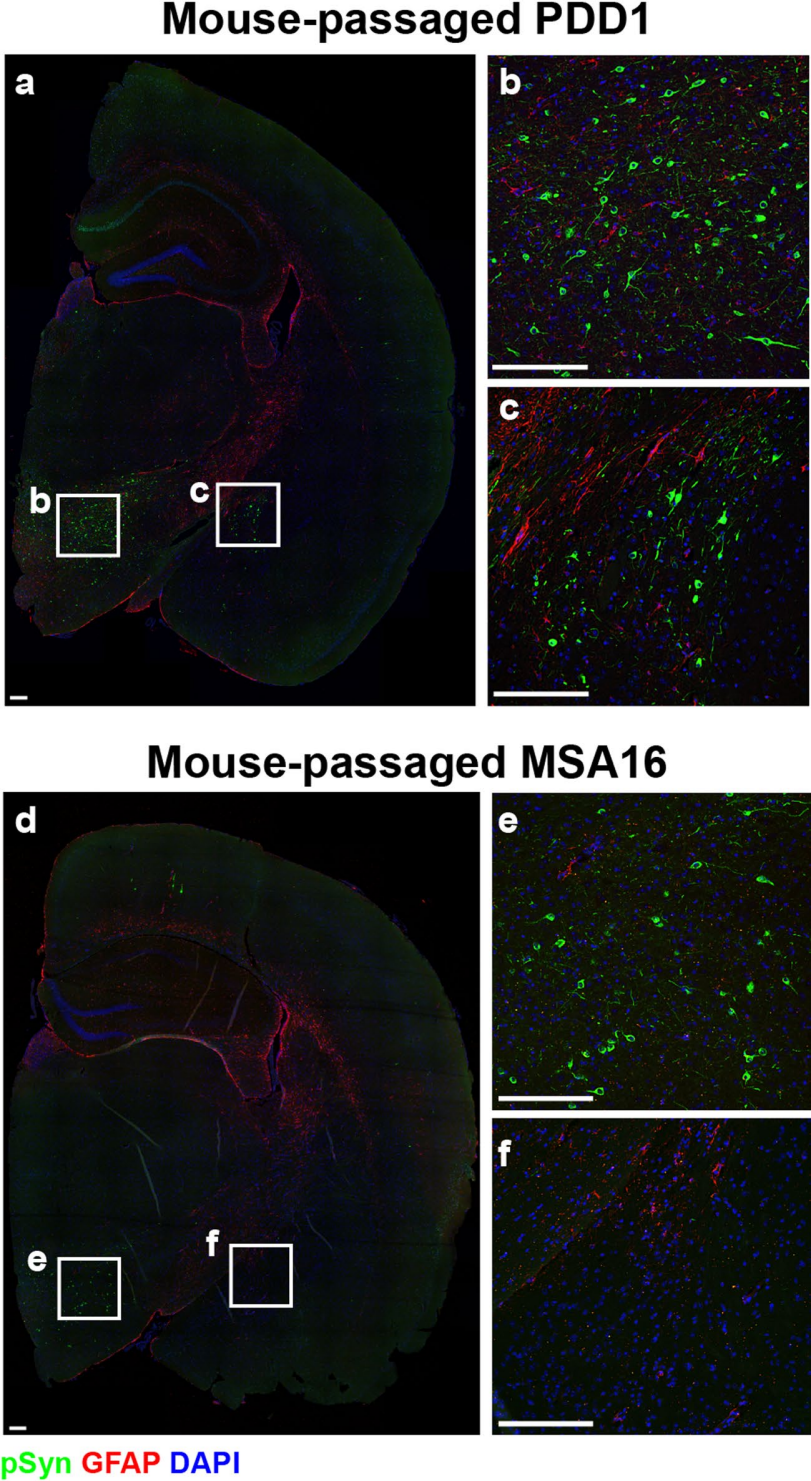
Mouse-passaged PDD1 induces robust phosphorylated α-synuclein pathology in the hypothalamus and amygdala of TgM83^+/-^ mice. TgM83^+/-^ mice were intracranially (i.c.) inoculated with either 30 µL of mouse-passaged PDD1 or MSA16 (reported previously in [29]) patient samples. Following onset of neurological signs, formalin-fixed half-brains were analyzed for phosphorylated α-synuclein pathology (EP1536Y primary antibody, 1:10,000). (**a**) Representative image of phosphorylated α-synuclein pathology in a TgM83^+/-^ mouse inoculated with mouse-passaged PDD1. Insets show inclusions in the (**b**) hypothalamus and (**c**) amygdala. (**d**) Representative image of phosphorylated α-synuclein pathology in a TgM83^+/-^ mouse inoculated with mouse-passaged MSA16. Insets show inclusions in the (**e**) hypothalamus and (**f**) amygdala. Phosphorylated α-synuclein in green, glial fibrillary acidic protein (GFAP; 1:500) in red, and 4’,6-diamidino-2-phenylindole (DAPI) in blue. Scale bar: 200 μm

Neuropathological analysis of secondary passage of PDD1 found significant phosphorylated α-synuclein inclusions in the hindbrains of animals injected with homogenate from both Mouse B and Mouse E compared to [C2] (*P* < 0.05), as well as spread to some forebrain regions (Fig. 3b). We then compared the inclusions found in mice inoculated with passaged PDD1 (Fig. 4a) and passaged MSA16 patient samples (Fig. 4d). In doing so, we found abundant α-synuclein inclusions in more forebrain regions, including the hypothalamus (HTH; Fig. 4b) and amygdala (Fig. 4c). While it is common to see some deposition in the HTH of MSA-inoculated TgM83^+/-^ mice (Fig. 4e), pathology in the amygdala is more variable. As is shown in Fig. 4c and f, mice inoculated with passaged PDD1 developed several inclusions within the amygdala, whereas the representative image from a mouse inoculated with passaged MSA16 shows no amygdala pathology. Neuropathology from mice inoculated with passaged MSA16 was previously reported [29]. Combining the subtle but important differences in incubation period, gait abnormalities, and neuropathological inclusions suggested that there may be differences between the α-synuclein strain in MSA patients and the strain isolated by passage of patient sample PDD1.

### Isolation of a novel α-synuclein strain following passage of patient sample PDD1

To assess the strain properties of the passaged PDD1 sample, we characterized the infectivity of α-synuclein prions isolated from both primary and secondary passage in our α-syn140–YFP biosensor cell assay panel [25]. We previously showed that this panel of cell lines has higher resolution than other methods for differentiating between α-synuclein strains and is a powerful tool for investigating strain adaptation following in vivo transmission [29]. In this recently established panel of cell lines, we determined that a biologically meaningful infection occurs when samples induce an increase in measured fluorescence by at least 2 × 10^5^ arbitrary units (A.U.). Here, brains from all symptomatic animals from primary passage of PDD1 (Mice B, D, E, and F; Fig. 1b), as well as passaged C2, were tested for infectivity in cells expressing the following mutations: A30G, E46K, K80E, G51D, A53E, A53T, V55Y, V66F, and V74P (Table 4). Compared to α-synuclein isolated from the brains of mice inoculated with patient sample C2, α-synuclein isolated from primary passage of PDD1 failed to induce aggregation in cell lines expressing the A30G mutation. As noted above, while the E46K mutation is known to cause familial LBD [35], we previously demonstrated that this mutation inhibits MSA propagation in vitro [28] and in vivo [36]. More recently, we showed that this inhibitory effect is due to disruption of an essential salt bridge between residues E46 and K80 [25]. Consistent with MSA strain biology, we found that the PDD1 primary passage samples were unable to infect either the E46K or the K80E cell lines. We also found that the samples failed to induce aggregate formation in cells expressing the G51D and A53E mutations. However, as shown in Fig. 1, the samples did replicate in cells expressing the A53T mutation. We also found that the PDD1 primary passage samples infected the V55Y and the V66F cell lines, but the V74P mutation inhibited replication.

**Table 4:** Cell infection using samples from primary passage of patient sample PDD1

Mutation	Passaged C2^a^ (Ave ± SD × 10^5^ A.U.)	Passaged PDD1^b^ (Ave ± SD × 10^5^ A.U.)	*P* value
A30G	0.4 ± 0.3	1.2 ± 1.0	0.13
E46K	0.0 ± 0.0	0.1 ± 0.1	0.72
K80E	0.0 ± 0.0	0.0 ± 0.0	0.83
G51D	3.6 ± 1.6	3.0 ± 0.5	0.45
A53E	1.1 ± 0.3	1.2 ± 0.8	0.97
A53T	5.7 ± 2.0	38 ± 19	0.003
V55Y	1.2 ± 0.3	4.6 ± 1.9	0.003
V66F	2.6 ± 0.4	13 ± 1.7	< 0.0001
V74P	0.3 ± 0.2	0.4 ± 0.2	0.25

^a^Data reported from 6 technical replicates tested from brain homogenate used as inoculum

^b^Data reported from 4 biological replicates

Intriguingly, we did not observe the same strain properties when we tested brains from the secondary passage of patient sample PDD1 using either Mouse B or Mouse E as our starting inoculum (Table 5). Secondary passage of C2 did not infect any cell line we tested, however, samples from secondary passage of PDD1 did infect cells expressing the A30G mutation. Similar to the primary passage samples, the E46K and the K80E mutations blocked biologically relevant infection. However, samples from both groups of secondary passaged PDD1 mice significantly infected cell lines harboring the G51D, A53E, A53T, V55Y, V66F, and V74P mutations (Table 5).

**Table 5:** Cell infection using samples from secondary passage of patient sample PDD1

Mutation	[C2] (Ave ± SD × 10^5^ A.U.)	[Mouse B] (Ave ± SD × 10^5^ A.U.)	[Mouse E] (Ave ± SD × 10^5^ A.U.)
A30G	0.3 ± 0.2	8.6 ± 5.0, *P* < 0.0001	6.2 ± 3.5, *P* < 0.0001
E46K	0.1 ± 0.1	0.1 ± 0.1, *P* = 0.35	0.4 ± 0.7, *P* = 0.26
K80E^a^	0.0 ± 0.0	0.0 ± 0.0, *P* = 0.02	0.0 ± 0.0, *P* = 0.0003
G51D	2.9 ± 0.7	9.4 ± 4.4, *P* = 0.0004	5.1 ± 2.5, *P* = 0.02
A53E	0.9 ± 0.3	4.4 ± 3.4, *P* = 0.005	6.0 ± 4.3, *P* = 0.001
A53T	5.3 ± 1.4	38 ± 18, *P* < 0.0001	44 ± 15, *P* < 0.0001
V55Y	1.6 ± 0.8	7.4 ± 3.7, *P* = 0.0002	6.4 ± 2.8, *P* < 0.0001
V66F	5.2 ± 1.5	15 ± 6.1, *P* = 0.0002	14 ± 9.8, *P* = 0.02
V74P	0.4 ± 0.2	2.5 ± 1.4, *P* = 0.0002	2.0 ± 1.0, *P* = 0.0001

^a^When rounded to two significant figures, small differences between sample groups are no longer apparent

This noticable change in the infectivity profile from primary to secondary passage suggests that further adaptation or strain selection of the original α-synuclein strain in the PDD1 patient sample occurred during secondary passage studies. To confirm our inability to detect this substrain in the starting patient sample, we would ideally compare the cell assay data from the passaged samples with the starting inoculum. However, an insufficient amount of remaining tissue from the substantia nigra hampered this direct comparison. Instead, we assayed α-synuclein prions isolated from the basal ganglia from patient PDD1 in the same panel of cell lines and found that the sample was unable to replicate in any cell line tested (Table 6). When we then compare this increase in infectivity profile observed during serial passage of patient sample PDD1 with the reduction in α-synuclein bioreporter cell line infectivity seen with serial passage of patient sample MSA16, our data indicate that we have isolated a novel α-synuclein strain from patient sample PDD1 that is experimentally distinct from MSA or mouse-passaged MSA (Table 7).

**Table 6:** Cell infection using basal ganglia from patient PDD1

Mutation	PDD1 (Ave ± SD × 10^5^ A.U.)
A30G	1.2 ± 0.8
E46K	0.1 ± 0.0
K80E	0.0 ± 0.0
G51D	1.8 ± 3.3
A53E	0.8 ± 0.4
A53T	9.2 ± 3.8
V55Y	1.0 ± 0.2
V66F	3.4 ± 0.5
V74P	0.4 ± 0.2

**Table 7:** Infectivity profile of patient samples MSA16 and PDD1 before and after passage in TgM83^+/-^ mice

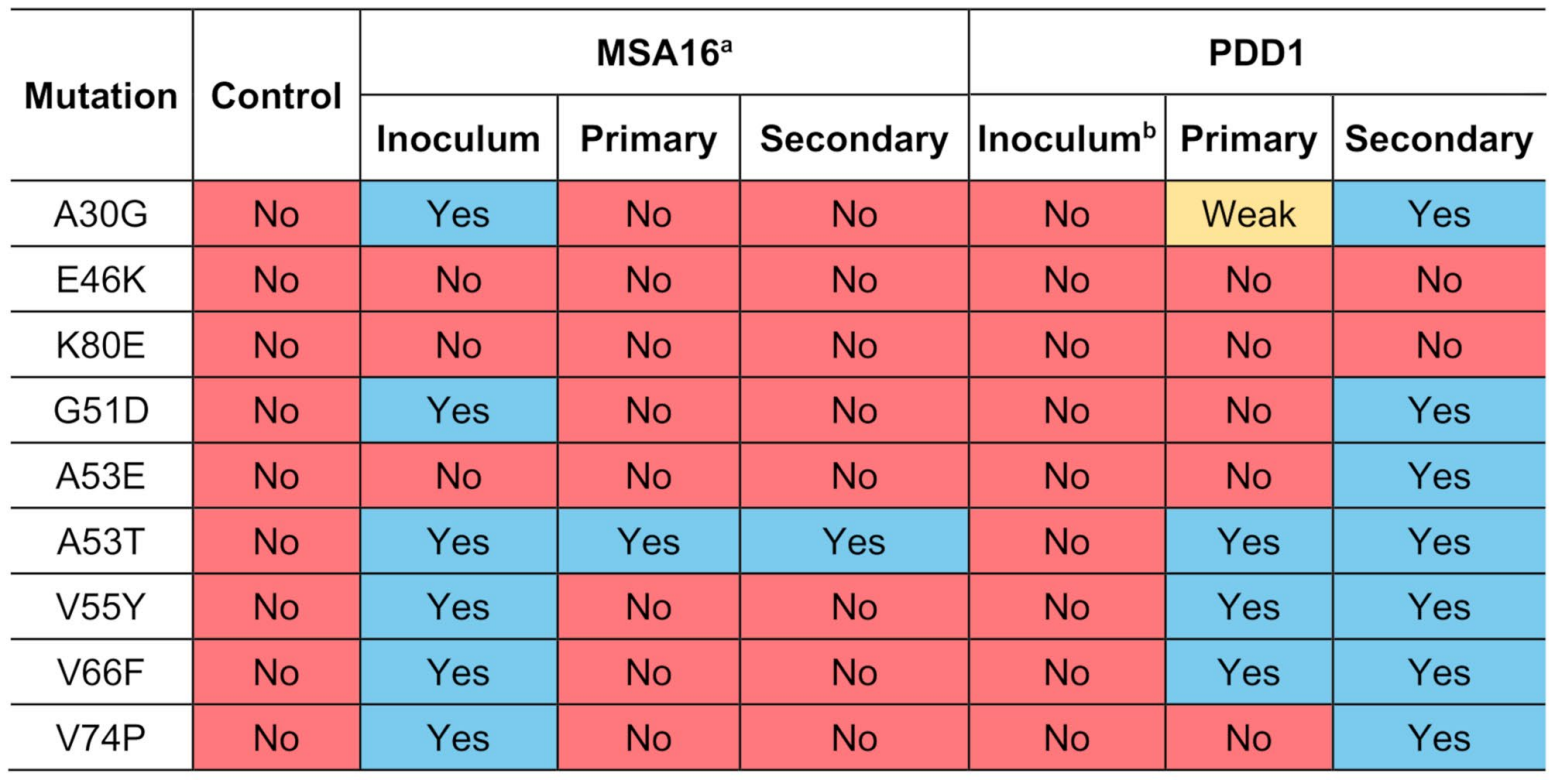	

^a^Data published previously in [29]; Red, no infection; yellow, weak infection; blue, infection

^b^Data from the basal ganglia of patient PDD1, except for the E46K and A53T data, which were from direct assay of the substantia nigra starting inoculum

**Fig. 5:**
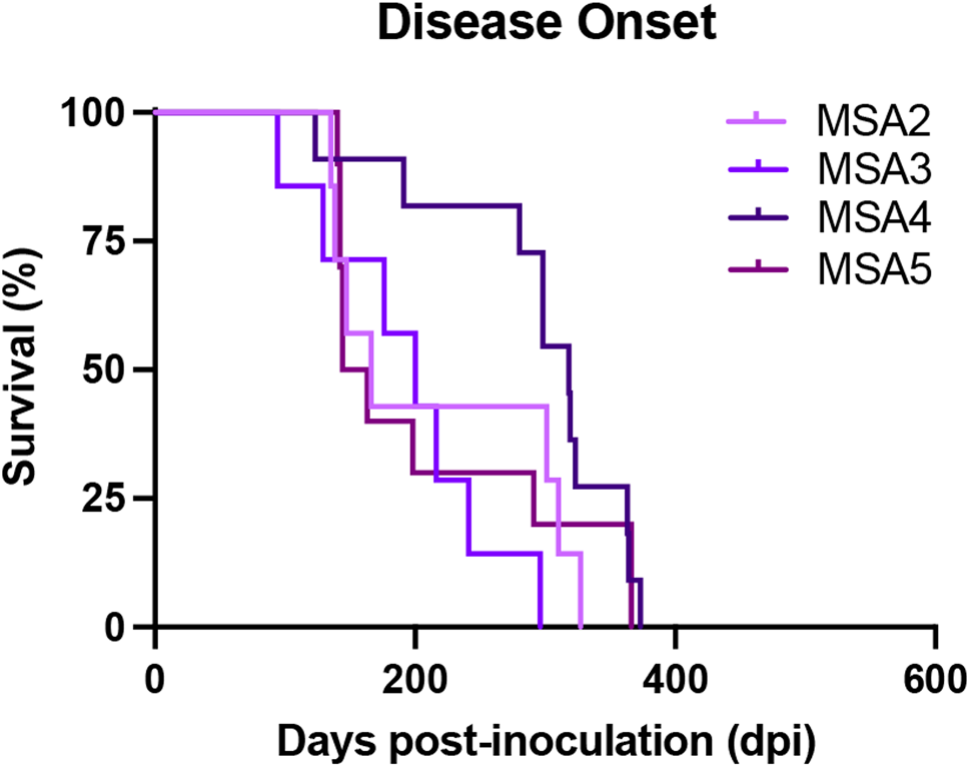
MSA transmission to TgM83^+/-^ mice. TgM83^+/-^ mice were intracranially inoculated with 30 µL of brain homogenate standardized to 1 mg/mL total brain from one of four MSA patient samples (MSA2, MSA3, MSA4, and MSA5). Mice inoculated with MSA2 developed neurological signs in 210 ± 86 days post-inoculation (dpi). Mice inoculated with MSA3 developed neurological disease in 205 ± 77 dpi. Mice inoculated with MSA4 developed disease in 295 ± 76 dpi. Mice inoculated with MSA5 developed clinical disease in 210 ± 95 dpi

Finally, to further support this interpretation of our findings, we compared the cell infectivity profile of four additional MSA patient samples in our α-synuclein bioreporter cell lines, along with brains collected from terminal TgM83^+/−^ mice inoculated with the same patient samples. Patient samples MSA2, MSA3, MSA4, and MSA5 all induced terminal disease in TgM83^+/−^ mice with an average incubation period ranging from 205 to 295 dpi (Fig. 5). Following disease onset, we isolated α-synuclein prions from frozen half-brains collected from the terminal mice, which we compared with the starting inoculum using our α-syn-YFP cell lines (Table 8). While variability in the cell infectivity profile between MSA patient samples exists, as we reported previously [25], consistent with our findings using patient sample MSA16, there is a notable decrease in cell infectivity following primary passage. For example, MSA patient samples consistently lose the ability to replicate in cells expressing the G51D and A53E mutations, and show reduced replication in the A30G, V55Y, and V66F cell lines. Altogether, our data indicate that the α-synuclein strain isolated from serial passage of patient sample PDD1 is not MSA.

**Table 8:** Cell infection using MSA patient samples and passaged MSA samples

Cell Line	MSA2	[MSA2]	MSA3	[MSA3]	MSA4	[MSA4]	MSA5	[MSA5]
A30G	15 ± 5.0	6.6 ± 4.6	11 ± 5.9	4.0 ± 3.5	0.3 ± 0.1	2.4 ± 3.8	6.7 ± 2.1	9.8 ± 9.0
E46K	2.3 ± 1.1	0.4 ± 0.2	1.0 ± 0.3	0.6 ± 0.7	0.7 ± 0.3	0.7 ± 0.3	0.7 ± 0.2	0.6 ± 0.3
K80E	0.0 ± 0.0	0.0 ± 0.0	0.0 ± 0.0	0.0 ± 0.0	0.0 ± 0.0	0.0 ± 0.0	0.0 ± 0.0	0.0 ± 0.0
G51D	31 ± 11	5.7 ± 2.0	21 ± 7.6	12 ± 6.1	2.7 ± 1.2	4.9 ± 2.0	6.0 ± 2.6	4.8 ± 2.2
A53E	7.3 ± 3.0	2.8 ± 1.6	5.2 ± 1.5	3.0 ± 2.1	2.5 ± 1.1	3.5 ± 2.0	1.9 ± 0.5	3.0 ± 1.2
A53T	100 ± 23	50 ± 18	81 ± 17	62 ± 12	35 ± 8.9	37 ± 21	39 ± 6.8	51 ± 20
V55Y	23 ± 3.8	11 ± 3.4	27 ± 5.8	16 ± 5.0	7.9 ± 5.0	9.0 ± 4.4	10 ± 2.6	12 ± 5.2
V66F	26 ± 5.0	12 ± 2.6	19 ± 4.3	12 ± 3.4	10 ± 4.0	10 ± 3.1	18 ± 3.6	13 ± 5.2
V74P	2.2 ± 0.9	1.0 ± 0.5	1.8 ± 0.5	1.2 ± 1.0	0.9 ± 0.3	1.4 ± 0.9	1.1 ± 0.7	1.5 ± 0.8

Data reported as average ± standard deviation × 10^5^ A.U

## Discussion

Several groups including ours have explored the transmissibility of α-synuclein strains isolated from synucleinopathy patient samples with varying levels of success. Evidence has increasingly demonstrated that MSA patient samples are highly transmissible to cellular and rodent models, whereas LBD patient samples typically fail to replicate in vitro or cause clinical disease in vivo without additional sample manipulation. Because of this, we were surprised to discover that inoculation of patient sample PDD1 into TgM83^+/-^ mice resulted in neurological disease in four out of six animals over an extended incubation period (Fig. 1a; Table 3). Our initial investigation of brain tissue from the affected mice, using cell assay and neuropathology, suggested that the α-synuclein strain we isolated was more consistent with the MSA strain than the LBD strain. Revisiting the autopsy report confirmed that the patient diagnosis of PDD was consistent with both the clinical and neuropathological features reported. However, reassessment of the diagnostic slides identified the presence of oligodendroglial staining in the substantia nigra, along with several additional brain regions including the pons, medulla, and amygdala (Fig. 2). This finding is consistent with recent studies by some of the authors demonstrating that glial inclusions are more common than was previously thought [37].

While LBs and GCIs are the predominant pathological hallmarks of LBDs and MSA, respectively, potential GCI-like co-pathology has only been noted either in a few cases of idiopathic PD with no genotyping data [38, 39] or in familial PD associated with the *SNCA* mutations G51D and A53E [40, 41]. However, recent studies reported by Lau et al. investigating the transmissibility of two G51D PD patient samples found that i.c. inoculation of TgM83^+/-^ mice with these samples caused a restricted subclinical infection [32], rather than the terminal neurological signs seen here with PDD1 inoculation. Additionally, while transmission studies using A53E patient samples have not been reported, our previous work propagating MSA patient samples in α-syn140–YFP cell lines found that α-synuclein from MSA patient samples cannot replicate in cells expressing the A53E mutation [25]. This is also consistent with data reported here, showing that the A53E mutation largely impeded replication of MSA and mouse-passaged MSA in vitro (Table 8). Together, these findings suggest that the glial pathology found in the G51D and A53E mutation carriers is unlikely to be representative of true GCI co-pathology. The α-synuclein found in these patients is clearly a distinct strain from that present in patient sample PDD1. Moreover, given that only two studies have shown that PD samples can induce subclinical infection with limited neuropathology in mice [31, 32], we thought it unlikely that our studies were detecting transmission of the LBD strain. Instead, we hypothesized that our data likely reflected transmission of a low-titer MSA strain present as a co-pathology in the PDD1 patient sample.

To test this hypothesis, we performed secondary transmission studies using brain homogenates from two symptomatic mice in the primary injection group (Mice B and E). Both cohorts showed a shortening of the incubation period from 390 ± 141 dpi to 125 ± 32 dpi (Mouse B) and 157 ± 35 dpi (Mouse E; Fig. 3a). Notably, this incubation period is still slightly longer than typical passaged MSA (93 ± 24 dpi for the MSA16 data discussed here). Additionally, we observed extensive phosphorylated α-synuclein deposition within the hindbrain that spreads into some forebrain regions (Figs. 3b and 4), with notable differences from what we typically see with MSA transmissions. For example, we observed immunostaining in approximately 7% of the Mid following second passage of PDD1, whereas our recent study using second passage MSA induced neuropathology in only 2% of the Mid in terminal mice [29]. Consistent with this difference, we also found dense deposition of phosphorylated α-synuclein in the amygdala of mouse-passaged PDD1 samples when compared to our passaged MSA cohorts, where amygdala pathology is more variable (Fig. 4). These data, along with minor differences in gait abnormalities at onset of clinical signs, provided the first suggestion that the α-synuclein strain isolated from the PDD1 patient sample may be distinct from the α-synuclein strain present in MSA.

We recently showed that our robust panel of α-syn140–YFP cell lines [25] can discriminate subtle differences between α-synuclein strains when other approaches (such as biochemical stability) cannot [29]. Using this assay, we investigated the strain biology of the α-synuclein prions isolated from both primary and secondary passage of patient sample PDD1 (Tables 4 and 5). Our prior work has shown that both the E46K and K80E mutations disrupt a critical salt bridge in the MSA fibril structures [42] and block MSA propagation in both cell and mouse models of disease [25, 28, 36]. Consistent with these data, α-synuclein from both primary and secondary passage of PDD1 was also unable to replicate in the E46K and K80E cell lines, suggesting that an E46/K80 salt bridge is likely present in the PDD1-derived fibril structure. Interestingly, this salt bridge is seen across several α-synuclein fibril structures (reviewed in [36]) but is notably absent from the LBD fibril fold in which a salt bridge between E35/K80 stabilizes the conformation instead [24]. These data suggest that the α-synuclein strain identified here is not the previously reported LBD strain. It is tempting to hypothesize that the strain isolated here is the Lewy-MSA hybrid strain recently reported by Enomoto et al., given that the E46/K80 salt bridge is preserved in the reported structures [43], however, major differences in the neuropathological inclusions identified in the patient tissue between the two patients suggests this is unlikely to be the case.

**Fig. 6:**
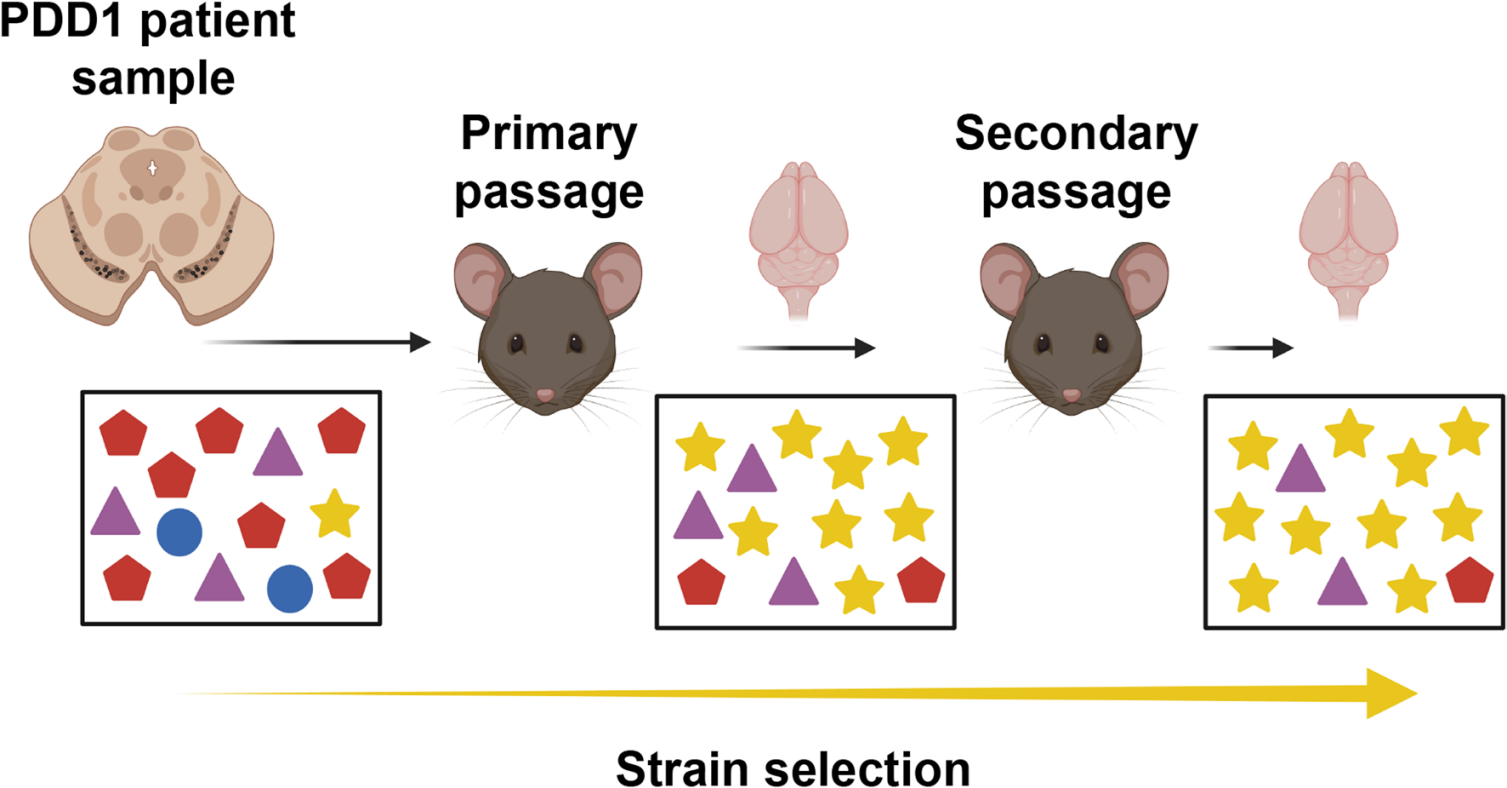
Schematic of α-synuclein strain selection during mouse passage. A patient sample can contain multiple different conformations of a misfolded protein, with one particular structure emerging as the dominant strain in disease. We posit that the substantia nigra from patient sample PDD1 likely contains multiple conformations, or strains, of α-synuclein, which are depicted here as red pentagons, purple triangles, blue circles, and gold stars. One of these strains (red pentagons) was likely the predominant conformation in patient PDD1 and is most responsible for the clinical presentation of disease. However, primary passage of the patient sample into TgM83^+/-^ mice selected for a subset of α-synuclein structures that are compatible with the new replication environment. This is shown here by the lower abundance of red pentagons and loss of blue circles paired with the emergence of the gold stars. Secondary passage in mice allowed continued strain selection to occur, with the gold stars becoming the predominant strain within the mouse brain. This selection is seen experimentally as the infectivity profile of the isolated strain expands during primary and secondary mouse passage relative to the starting inoculum. This figure was made using Biorender.com

Aside from the E46K and K80E mutations, samples from the secondary passage of PDD1 infected the other seven cell lines we tested. By comparison, the primary passage samples could only significantly replicate in cells expressing the A53T, V55Y, and V66F mutations. This change in infectivity profile between mouse passages is likely reflective of α-synuclein conformational selection, leading to our hypothesis that the PDD1 patient sample contains a mixture of α-synuclein conformations, a subset of which were selected during primary passage and then further enriched during secondary passage (Fig. 6). Several studies on prion disease have shown that mixtures of PrP^Sc^ strains can occur in both animal and human prion diseases. Moreover, a subset of strains can be selected for during passage into a new species for multiple reasons including substrate compatibility [44–46]. Similarly, our data suggest that despite the higher abundance of the LBD strain, which does not transmit disease to TgM83^+/-^ mice, in patient sample PDD1, the presence of a less abundant α-synuclein conformation in the substantia nigra (gold star in Fig. 6) enabled disease transmission to occur when the less abundant strain emerged in the new replication environment. Further selection and isolation of this strain occurred during secondary passage, leading to the expansive infectivity profile seen when we used the second passage PDD1 samples in our cell assay, particularly in comparison to the starting profile for patient sample PDD1 (Table 7). Moreover, these data are strikingly different compared to the infectivity profile of MSA prions before and after passage (Tables 7 and 8, and [29]), clearly demonstrating that the α-synuclein strain isolated from patient sample PDD1 is not the MSA strain.

It is important to remember that even though we tend to limit our investigation of neurodegenerative diseases to a single protein, these disorders often exhibit co-pathology of multiple misfolded proteins at autopsy. For example, α-synuclein in LBs and tau fibrils co-occur in the brains of up to 50% of patients with Alzheimer’s disease [47–49], tau pathology is documented in nearly half of patients with PD [50–52], and β-amyloid plaques are present in nearly 40% of patients with PD and 60–80% of patients with PDD or DLB [53]. However, the possibility of a co-pathology arising from a single protein, such as α-synuclein, is likely underappreciated. Research on PrP^Sc^ strains in patients with Creutzfeldt-Jakob disease (CJD) has shown that multiple strains can coexist in individual patients with CJD, which has unique consequences for disease pathogenesis and treatment [54–56]. For example, while no data specific to PrP^Sc^ strain interactions in CJD cases have been reported, many investigators have experimentally demonstrated that two PrP^Sc^ strains can compete, or interfere, with one another when injected into a single host [57–61]. A dominant strain may suppress, but not eliminate, any additional strains present within a cell or brain. In this scenario, a faster replicating α-synuclein strain (such as MSA) could suppress a strain with slower kinetics (such as LBD) to cause disease in a patient. It is also possible that two strains may even coexist to produce a mixed clinical phenotype of disease; this may be the case for the patient with PDD studied here, given that the patient exhibited mixed phenotypic clinical signs along with the oligodendroglial inclusions. In support of this hypothesis, multiple studies report mixed phenotypic clinical signs in cases of “minimal change” MSA in which patients present with parkinsonian symptoms due to neuronal loss in key regions, such as the substantia nigra, while also developing GCI and neuronal cytoplasmic inclusion pathology throughout the brain [62–64]. Though these patients do not exhibit co-pathologies, it is possible that this patient with PDD is a rare case resulting from two distinct synucleinopathies interacting with one another. Moreover, the recent identification of a Lewy-MSA hybrid strain clearly indicates the presence of additional novel α-synuclein strains that we are only now starting to identify [43].

A secondary consequence of co-pathologies from the same protein is the potential emergence of a drug-resistant strain. For example, if an effective therapeutic successfully targets LBD pathology but not GCI-like pathology in a patient such as PDD1, the MSA symptoms would likely worsen because the MSA strain can continue replicating and spreading without interference from the LBD strain. This phenomenon has been seen with both PrP and yeast prions [65–68]. Experimental treatment of PrP^Sc^ with either the 2-aminothiazole IND24 or swainsonine resulted in the emergence of drug-resistant PrP^Sc^ strains. While removal of drug treatment restored the susceptibility of PrP^Sc^ strains in both cases [66, 67, 69], the recognition that drug resistance can and does occur in the presence of prion strain mixtures has profound impacts on the design and development of meaningful clinical interventions for all patients with neurodegenerative disease.

In summary, this is the first report of a novel α-synuclein strain isolated from the substantia nigra of a synucleinopathy patient, though the partial transmission of PD samples to TgM83^+/-^ mice previously reported by Lavenir et al. [22]. suggests the presence of this strain in LBD patient samples could be more prevalent in the patient population. Despite the robust presence of LBs along with oligodendroglial in the PDD patient sample, the α-synuclein strain isolated by serial passage in TgM83^+/-^ mice exhibits unique properties distinct from either the LBD or MSA strains, including an ability to increasingly replicate in cultured cells expressing mutant α-synuclein. While these findings are currently limited to a single patient sample, a better understanding of how α-synuclein co-pathologies interact is needed to inform the design of effective diagnostics and therapeutics for patients with neurodegenerative disease.

## Data Availability

Data is provided within the manuscript.
